# Prescription of topiramate to treat alcohol use disorders in the Veterans Health Administration

**DOI:** 10.1186/1940-0640-8-12

**Published:** 2013-07-08

**Authors:** A C Del Re, Adam J Gordon, Anna Lembke, Alex HS Harris

**Affiliations:** 1 Center for Health Care Evaluation, VA Palo Alto Health Care System, 795 Willow Rd, Menlo Park, CA 94025, USA; 2 Stanford University School of Medicine, 401 Quarry Road, Stanford, CA 94305, USA; 3 VA Pittsburgh Healthcare System & University of Pittsburgh School of Medicine, 7180 Highland Drive (151 C-H), Pittsburgh, PA 15206-1206, USA; 4 Stanford Department of Psychiatry and Behavioral Sciences, Stanford University School of Medicine, 401 Quarry Road, Stanford, CA 94305, USA

**Keywords:** Alcohol use disorders, Addiction, Pharmacotherapy, Topiramate, Pharmacotherapy utilization, Veterans

## Abstract

**Background:**

As a quality improvement metric, the US Veterans Health Administration (VHA) monitors the proportion of patients with alcohol use disorders (AUD) who receive FDA approved medications for alcohol dependence (naltrexone, acamprosate, and disulfiram). Evidence supporting the off-label use of the antiepileptic medication topiramate to treat alcohol dependence may be as strong as these approved medications. However, little is known about the extent to which topiramate is used in clinical practice. The goal of this study was to describe and examine the overall use, facility-level variation in use, and patient -level predictors of topiramate prescription for patients with AUD in the VHA.

**Methods:**

Using national VHA administrative data in a retrospective cohort study, we examined time trends in topiramate use from fiscal years (FY) 2009–2012, and predictors of topiramate prescription in 375,777 patients identified with AUD (ICD-9-CM codes 303.9x or 305.0x) treated in 141 VHA facilities in FY 2011.

**Results:**

Among VHA patients with AUD, rates of topiramate prescription have increased from 0.99% in FY 2009 to 1.95% in FY 2012, although substantial variation across facilities exists. Predictors of topiramate prescription were female sex, young age, alcohol dependence diagnoses, engagement in both mental health and addiction specialty care, and psychiatric comorbidity.

**Conclusions:**

Veterans Health Administration facilities are monitored regarding the extent to which patients with AUD are receiving FDA-approved pharmacotherapy. Not including topiramate in the metric, which is prescribed more often than acamprosate and disulfiram combined, may underestimate the extent to which VHA patients at specific facilities and overall are receiving pharmacotherapy for AUD.

## Background

Four medications have been approved by the US Food and Drug Administration (FDA) for the treatment of alcohol dependence, including naltrexone (both oral and injectable extended release), acamprosate, and disulfiram [1]. Naltrexone is an opioid antagonist that reduces the reward properties of and cravings for alcohol [2]. Acamprosate reduces cravings and has been found to be efficacious in maintaining, but perhaps not producing, abstinence [3]. While disulfiram may be more familiar to clinicians than naltrexone or acamprosate, some clinical practice guidelines do not recommend disulfiram as a first-line pharmacological treatment because of significant toxicity risks and limited evidence of effectiveness [4].

Utilization rates for the four FDA-approved medications are generally low [1]. Of the approximately 19 million patients meeting criteria for alcohol dependence in the United States, less than 150,000 are treated with FDA-approved pharmacotherapy [5,6]. In 2009, among patients of the US Veterans Health Administration (VHA), the largest health-care system in the United States, 4.7% of veterans with diagnosed alcohol dependence (3.4% with any alcohol use disorder [AUD]) filled at least one prescription for naltrexone, acamprosate, or disulfiram [1]. As with other classes of psychiatric medications, such as antidepressants, each of the medications for AUD has particular strengths, contraindications, and side effects; therefore, a specific patient may benefit more from one medication compared with another. Ideally, patients should have access to as many efficacious medications for AUD as possible so they can work with their health-care providers to find the agent that maximizes benefits while minimizing side effects.

Several medications, other than the four approved medications for alcohol dependence, have shown promise in reducing alcohol consumption. Perhaps the most impressive evidence exists for the antiepileptic medication topiramate, shown to reduce alcohol consumption [7-9] with clinical effect sizes that are at least as large as naltrexone and acamprosate [10]. Johnson and colleagues found that 300 mg per day of topiramate reduced heavy drinking days 8.5% more than a placebo [8]. However, topiramate was also associated with certain side effects compared with placebo, including paresthesia, taste perversion, anorexia, and difficulty with concentration. A review of many studies of topiramate in the treatment of psychiatric disorders concluded that it is generally well-tolerated with the most common serious adverse events being paresthesia/numbness (12.9%), nausea/vomiting (6.2%), cognitive impairment (5.4%), headache (5.0%), and dizziness (5.0) [11].

Although not well understood, topiramate is thought to reduce craving for alcohol by targeting the glutamate brain pathways [12] and inhibiting dopamine release [9]. Interestingly, topiramate has also been found to be effective for other off-label uses, such as amelioration of obesity [13], eating disorders [14], and migraine headaches [15]. Of particular relevance, evidence suggests that topiramate has mood stabilizing properties [16] and is efficacious for treating certain psychiatric disorders, including bipolar disorder [11], borderline personality disorder [16], and post-traumatic stress disorder [17]. Alcohol dependence is often comorbid with these disorders; therefore, topiramate may be viewed by clinicians as a way to address multiple problems with one medication.

Although the utilization of FDA-approved pharmacotherapy for alcohol dependence in the VHA is known to be low and variable [18], nothing is currently known about the overall use of topiramate for alcohol dependence. Currently, VHA facilities are monitored regarding the extent to which patients with AUDs are receiving FDA-approved medications [19]. However, topiramate, which appears to be at least as effective as naltrexone, acamprosate, and disulfiram, is not included in current quality improvement metrics in the VHA or elsewhere [20]. Therefore, the extent to which VHA patients, at specific facilities or overall, are utilizing pharmacotherapy for AUDs may be underestimated. The goal of this study was to examine overall (and facility-level) prescription rates of topiramate for patients diagnosed with AUDs and time trends in the receipt of topiramate, as well as which patient characteristics are associated with greater likelihood of receipt of topiramate through prescription.

## Methods

### Calculating receipt of topiramate across four years

For each fiscal year (FY) (from October 1 through September 30, 2009–2012), we used the VHA National Patient Care Database (NPCD) to identify all veteran patients that had an AUD diagnosis (alcohol abuse or alcohol dependence, ICD-9-CM codes 303.9x or 305.0x) recorded in any outpatient or inpatient encounter during those years. Even though topiramate has primarily been studied in patients with alcohol dependence diagnoses, we included patients with alcohol abuse diagnoses due to the unreliability of this diagnostic distinction in clinical data and for consistency with the VHA quality improvement metrics, and because the abuse/dependence distinction has been eliminated in the revised Diagnostic and Statistical Manual of Mental Disorders (DSM-V) (http://www.dsm5.org). We then used the VHA Decision Support System (DSS) inpatient and outpatient pharmacy benefits datasets to determine the number of patients with AUD who filled a prescription for topiramate (defined as at least one VHA pharmacy record for topiramate) during the same year as their AUD diagnosis (either a new or ongoing diagnosis). From these data, we were able to calculate the annual rates of topiramate prescription among patient with AUD within the 141 VHA facilities nationwide.

### Patient characteristics predicting topiramate prescription

Using only the FY 2011 cohort—the most recent year for which all relevant data were available—we examined the patient-level predictors of topiramate prescription. From the NPCD we obtained data regarding gender, age (categorized as age <30, 30–55, >55), alcohol diagnosis (categorized as having an alcohol dependence diagnosis at any time during the year versus only an alcohol abuse diagnosis), receipt of mental health and/or addiction specialty care (categorized as “none,” “mental health,” “addiction,” and “both mental health and addiction”), and psychiatric comorbidities (categorized as “none,” “bipolar,” “depression,” “PTSD,” “PTSD and depression,” “schizophrenia,” and any other combination, classified as “other/multiple”) to determine their relationship with any topiramate prescription. The classification of psychiatric comorbidities into one of the seven mutually exclusive categories was based on previous research of topiramate for treating psychiatric illness [11] and on meeting a minimal percentage breakdown (>2% of total sample with particular psychiatric diagnosis required) into one of the mutually exclusive bins.

### Analytic plan

To describe and explore variability in facility-level rates of topiramate prescription, we calculated the rate of topiramate prescription (number of AUD patients who had at least one pharmacy record for topiramate divided by the total number of AUD patients in the facility) in FY 2011 for each of the 141 VHA facilities. All analyses predicting topiramate utilization at the patient level (0 = no, 1 = yes) were performed using generalized logistic mixed-effects models with a random effect for facility to account for the clustering of patients. We also conducted sensitivity analyses that explored the effect that the inclusion of patients diagnosed with migraine or seizure disorders may have had on our results. These sensitivity analyses included crosstabs of the number in our sample with either migraine or seizure disorders, the correlation between facility prescription rates of topiramate and FDA-approved medications for AUD (naltrexone, acamprosate, and/or disulfiram), and additional mixed-effects regression models that excluded patients diagnosed with migraine or seizure disorders. The mixed-effects regression models were conducted using the GLIMMIX function within the SAS statistical software (version 9.2), and graphics were produced with the ggplot2 package within the R statistical software (version 14.2). The VA Palo Alto Health Care System’s research office and Stanford University’s Human Research Protection Program approved this project.

## Results

Table 1 presents data on the percent of patients with AUD who received topiramate from FY 2009 through 2012. Approximately 10% of patients prescribed topiramate also received one of the other FDA-approved medications for AUD during the same fiscal year. In FY 2009, nearly 1% (n = 3736) of the 372,817 patients with an AUD filled a prescription for topiramate. This rate increased to 1.45% (n = 5270) in FY 2010 among 357,467 patients with AUD. In FY 2011, 1.45% (n = 5454) of all veterans with AUD (N = 375,777) filled a VHA pharmacy prescription for topiramate. In FY 2012, 1.95% (n = 7427) of all veterans with AUD (N = 381,815) filled a VHA pharmacy prescription for topiramate. Within the 141 VHA facilities in FY 12, the overall proportion of patients who received topiramate ranged from 0% to 6.5% (mean, 2.03%; median, 1.69%) with seven sites prescribing topiramate to more than 2% of patients with AUD. When restricting the analyses to patients who had contact with VHA addiction specialty care within the year, the facility-level proportions of patients who received topiramate ranged from 0% to 13.4% (mean, 2.97%; median, 2.47%) with 16 sites prescribing topiramate to more than 5% of patients with AUD.

**Table 1:** Patients who received topiramate for alcohol use disorders in the Veterans Health Administration, 2009–2012

**Year (total N**^**1**^**)**	**Topiramate prescription (n)**	**Topiramate prescription (%)**
2009 (N = 374,817)	3736	0.99
2010 (N = 357,467)	5270	1.45
2011 (N = 375,777)	5454	1.45
2012 (N = 389,242)	7427	1.95

^1^Total numbers represent unique patients with either alcohol abuse or dependence diagnoses as recorded in the yearly census of over five million patients.

**Figure 1:**
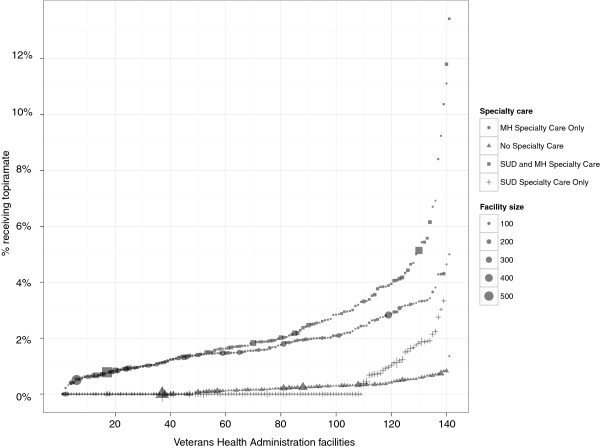
**Percentage of patients in 141 facilities receiving topiramate in FY 2011 by receipt of specialty care.** Displayed is the variability in facility level percentage of patients prescribed topiramate by specialty care services. The 141 VA facilities are ordered from smallest to largest percentage and the size of each point represents the number of patients within each respective specialty care service.

In the unadjusted single-predictor models, veteran patients with an AUD were more likely to fill a prescription for topiramate in FY 2011 if they were female, younger, had alcohol dependence, were involved in both mental health and addiction specialty care, and had a psychiatric comorbidity (Table 2). These results remained consistent in the adjusted multi-predictor analyses (Table 3). Specifically, patients were more likely to be prescribed topiramate if they were female (OR = 2.50; 95% confidence interval (CI), 2.31, 2.71), were between 30–55 years old compared with >55 (OR = 1.66; 95% CI, 1.56, 1.76), had alcohol dependence versus abuse (OR = 1.15; 95% CI, 1.08, 1.22), were involved in both mental health and addiction specialty care (OR = 3.75; 95% CI, 3.24, 4.43), and had a psychiatric comorbidity (depending on specific psychiatric diagnosis, estimates ranged from OR = 1.74; 95% CI, 1.43, 2.11 for schizophrenia to OR = 4.497; 95% CI, 4.07, 4.96 for other multiple psychiatric diagnoses). Figure 1 displays the facility prescription rates of topiramate by receipt of specialty care.

**Table 2:** Predictors of topiramate prescription and receipt of specialty care for alcohol use disorders in the Veterans Health Administration, fiscal year 2011

		**Topiramate (N = 5454)**	
**Variable**	**Total N**	**N**	**%**
Gender			
Male (reference)	360,434	4662	1.29
Female	15,231	792	5.20*
Age			
>55 (reference)	207,657	1904	0.92
30-55	143,805	3069	2.13*
<30	24,315	481	1.98*
Alcohol diagnosis			
Dependence (reference)	238,995	3892	1.63
Abuse	134,034	1540	1.15*
Specialty care			
None (reference)	105,549	248	0.23
Mental health	158,624	2798	1.76*
Addiction	10,532	52	0.49*
Both (mental health & addiction)	101,072	2356	2.33*
Psychiatric comorbidity			
None (reference)	188,762	897	0.48
Bipolar disorder	12,110	371	3.06*
Depression	42,296	748	1.77*
PTSD	61,665	1088	1.76*
PTSD & depression	35,570	1141	3.21*
Schizophrenia	9587	121	1.26*
Other/multiple	25,787	1088	4.22*

Note: * indicates p < .05 for the difference between the indicated group and the reference group in single predictor generalized linear mixed model with random effect for VHA facility.

**Table 3:** Multipredictor model predicting receipt of topiramate for alcohol use disorders in the Veterans Health Administration, fiscal year 2011

**Variable**	**Estimate**	**SE**	**t-value**	**OR [95%CI]**
Gender				
Female (reference: male)	0.92	0.04	22.09	2.50 [ 2.31, 2.71 ]
Age (reference: >55)				
30-55	0.51	0.03	16.22	1.66 [ 1.56, 1.76 ]
<30	0.32	0.05	6.01	1.38 [ 1.24, 1.53 ]
Alcohol diagnosis (reference: abuse)				
Dependence	0.14	0.03	4.21	1.15 [ 1.08, 1.22]
Specialty care (reference: no specialty care)				
Mental health	1.15	0.07	15.68	3.15 [ 2.73, 3.64 ]
Addiction	0.46	0.16	2.95	1.58 [ 1.17, 2.15]
Both (mental health & addiction)	1.32	0.08	17.67	3.75 [ 3.24, 4.43 ]
Psychiatric comorbidity (reference: none)				
Bipolar disorder	1.26	0.07	19.22	3.51 [3.09, 3.92]
Depression	0.78	0.05	14.86	2.19 [1.98, 2.43]
PTSD	0.98	0.05	20.08	2.66 [2.42, 2.93]
PTSD & depression	1.39	0.05	28.27	4.00 [3.63, 4.40]
Schizophrenia	0.55	0.10	5.56	1.74 [1.43, 2.11]
Other/multiple	1.50	0.05	30.11	4.49 [4.07, 4.96]
Intercept	−6.41	0.081	−79.45	0.002 [ 0.002, 0.002 ]

As topiramate can be prescribed for disorders unrelated to alcohol use, we also conducted a sensitivity analysis that explored the effect that the inclusion of patients diagnosed with migraine or seizure disorders may have had on our results. First, we tabulated the number in our sample with either migraine or seizure disorders (Table 4). As can be seen, 22.7% (n = 1239) of patients with an AUD and a prescription for topiramate also had a migraine or seizure disorder diagnosis. In addition, we determined which patients in our sample had contraindications for naltrexone, specifically acute hepatitis or a prescription of opioid pain medication. We found that, among those with a prescription for topiramate, 58% have contraindications for naltrexone, making it more plausible that the topiramate was being used to treat the AUD. Further, we examined the correlation between facility prescription rates of topiramate and FDA-approved medications for AUD (naltrexone, acamprosate, and/or disulfiram) and found a modest relationship (r < 0.20). Lastly, we reran the mixed-effects regression models analyses above excluding patients diagnosed with migraine or seizure disorders. The results of our models were virtually unchanged (i.e., some coefficients differed only at the third decimal place, but findings were unchanged).

**Table 4:** Crosstabs of topiramate prescription by migraine or seizure disorder diagnosis

	**Migraine or seizure disorder diagnosis**		
**Topiramate prescription**	**No**	**Yes**	**Totals**
No	357,865 (95.2%)	4215 (1.1%)	362,080 (96.4%)
Yes	12,458 (3.3%)	1239 (0.33%)	13,697 (3.6%)
Totals	370,323 (98.6%)	5454 (1.5%)	375,777 (100%)

Note: Total numbers for each cell are provided along with cell percentages in parentheses.

**Figure 2:**
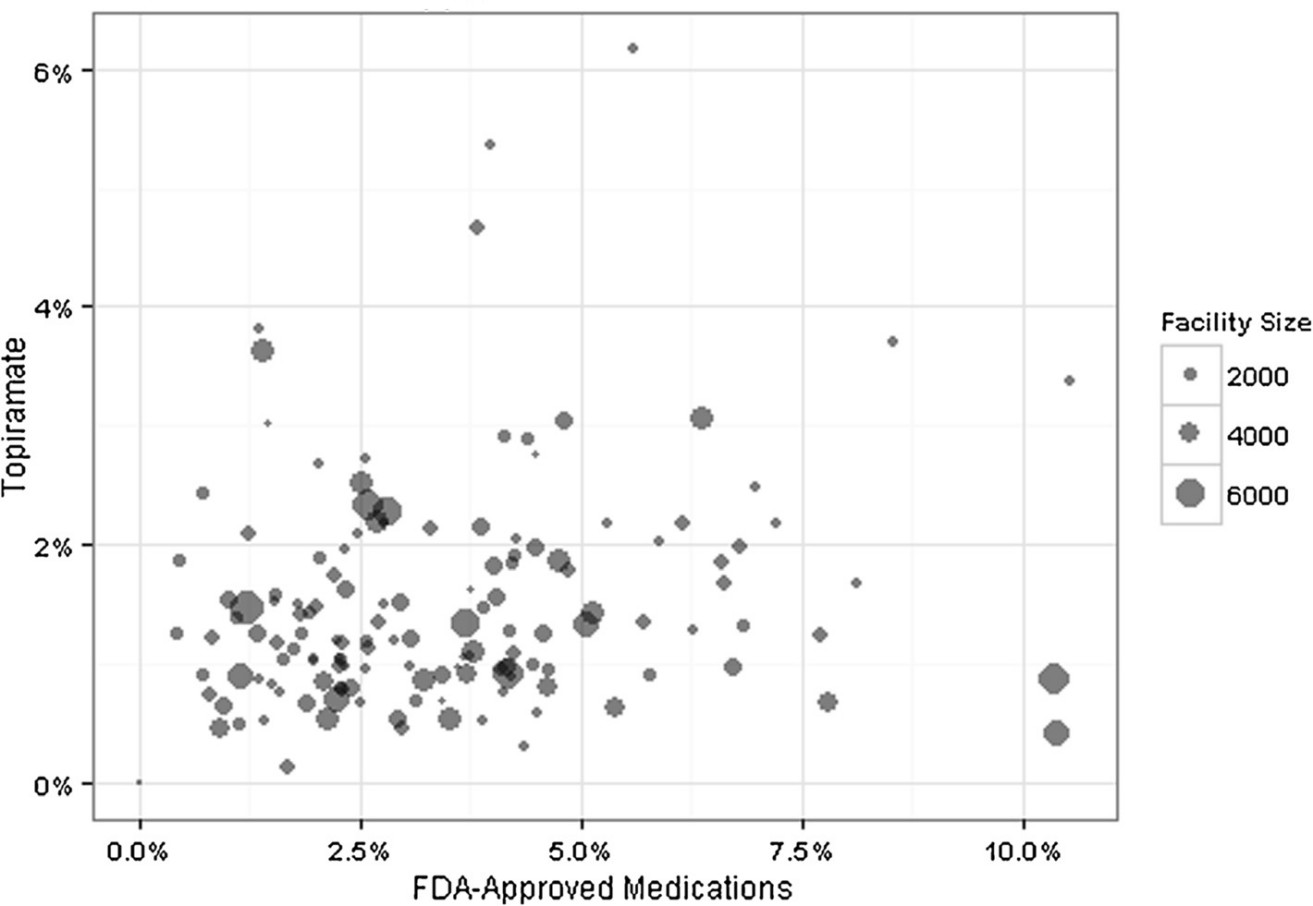
**Facility prescription rates of topiramate and FDA-approved medications for alcohol use disorders.** Displayed are the facility prescription rates of topiramate on the *y*-axis and FDA-approved medications for AUD on the *x*-axis.

Also, we conducted analyses to compare the number of patients with AUD who were prescribed topiramate with the number who were prescribed the FDA-approved medications in FY 2012. We determined that the 7427 patients receiving topiramate in FY 2012 was more than the number of patients receiving acamprosate (n = 1662), injectable naltrexone (n = 498), or disulfiram (n = 3455) but not as many as those receiving oral naltrexone (n = 9646) in patients with AUD. Figure 2 displays the facility prescription rates of topiramate and FDA-approved medications for AUD. Those facilities in the middle to upper-left quadrant of the figure (i.e., higher topiramate prescriptions and lower FDA-approved medication prescriptions) are those facilities likely to be penalized and receive lower performance ratings.

## Discussion

Although evidence supports the efficacy of topiramate for alcohol dependence [7-9,21-23], system monitoring metrics and quality measures of pharmacotherapy for AUD used within VHA [19] and developed elsewhere [24] only include FDA-approved medications (naltrexone, acamprosate, and disulfiram). This may not only underestimate actual overall patient utilization of pharmacotherapy for AUD but also unnecessarily penalize those facilities with higher rates of topiramate utilization.

We found that 1.95% of the nearly 400,000 patients treated in VHA facilities with a diagnosis of AUD were prescribed topiramate—more than acamprosate, injectable naltrexone, and disulfiram combined. Therefore, by not including topiramate in monitoring metrics, patient prescription of pharmacotherapy for AUD may be underestimated by as much as 40% in the VHA. It is worth noting that only about 10% of patients prescribed topiramate also were prescribed one of the other medications for AUD during the same fiscal year, meaning that the possible undercount is quite substantial. That said, the underestimation by 40% is the upper bound estimate due to limitations of the administrative data, which did not permit us to determine what topiramate was filled for specifically (e.g., prescription for something other than AUD).

Relatedly, those facilities with higher rates of topiramate prescriptions (versus FDA-approved pharmacotherapy) may falsely appear to have lower rates of pharmacotherapy use for AUD. This finding is strengthened by the fact that we found a modest relationship between facility prescription rates of topiramate and FDA-approved medications for AUD. The consequence of this finding is that some VHA facilities with higher rates of topiramate prescription for AUD may unjustifiably end up with lower AUD pharmacotherapy performance ratings than they would if topiramate was included in the specifications of these metrics.

At what point does a therapy become “evidence-based?” Currently, more than 10 studies (four of which were randomized controlled trials [RCTs]) have been conducted examining the efficacy of topiramate for alcohol dependence. A recent meta-analysis pooled the results of three placebo-controlled trials and reported that topiramate was more efficacious than placebo in terms of reducing the percent of heavy drinking days (23.2%; 95% CI, 15.7, 34.4) as well as increasing the number of abstinent days (mean difference, 2.9 days; 95% CI, 2.5, 3.3). The combined results of two other trials indicated that topiramate is also more efficacious than naltrexone [7]. Studies published since this meta-analysis provide further support for the efficacy of topiramate. One recent RCT of topiramate found those in the group with psychotherapy plus low-dose topiramate fared better at post-test and four-month follow-up than those allocated to psychotherapy alone (lower relapse rate in the topiramate group [66.7%] compared with psychotherapy alone [85.5%]) [25]. The strength of the evidence in support of topiramate for the treatment of alcohol dependence is at least on par with naltrexone and acamprosate and is stronger than that for disulfiram. However, because topiramate is now off-patent, it is extremely unlikely FDA approval will be sought for the treatment of alcohol dependence or any other new indications; a pharmaceutical company would be unlikely to seek a new indication for a medication through the costly FDA approval process if that medication is off-patent and not proprietary. The first quality metrics in this domain within the VHA and elsewhere [20] included only FDA-approved medications, even ones with poor evidence (i.e., disulfiram), perhaps because this strategy had high face validity and avoided more complicated judgments about which medications have sufficient supporting evidence. However, excluding medications with strong evidence but no FDA approval from quality metrics is short-sighted. Therefore, we encourage the inclusion of topiramate in related quality metrics and in quality improvement efforts intended to increase the active consideration of medications in the treatment of alcohol dependence.

### Patient-level predictors

Several patient characteristics were associated with greater likelihood of receipt of topiramate. Female patients with AUD were more likely than male patients to receive a prescription for topiramate (5.2% compared with 1.3%). This result is not unique to topiramate, as women, compared with men, are generally more likely to receive the other medications for alcohol dependence as well as specialty addiction treatment [1,26]. This association may be due to unobserved factors, such as women having greater likelihood of psychiatric diagnoses and treatment-seeking behavior compared with men [1].

Patients between age 30–55 were most likely (compared with those >55) to receive a prescription for topiramate. These results were similar to Harris et al's study of FDA-approved pharmacotherapy for AUD and may be related to older patients being less likely to receive any addiction treatment, including pharmacotherapy, due to cohort norms and stigmatization around treatment for alcohol dependence [1]. Another possible explanation for older patients receiving fewer prescriptions is that they have more medical comorbidities and, therefore, may be taking other medications that may be contraindicated for topiramate.

The rate of topiramate prescription was higher for patients diagnosed with alcohol dependence than for those diagnosed with alcohol abuse. This finding was expected and congruent with receipt patterns for the FDA-approved medications for alcohol dependence. Topiramate prescription rates were also higher for patients who received care in both VHA substance-use disorder and mental-health specialty treatment settings in the same year. Further, patients with psychiatric comorbidities were much more likely to have a prescription for topiramate (83% of patients with a prescription for topiramate had at least one psychiatric comorbidity) (Table 2). Topiramate has mood stabilizing properties [16] and is efficacious for treating several psychiatric disorders (bipolar disorder [11], borderline personality disorder [16], and post-traumatic stress disorder [17]). As AUD is often comorbid with psychiatric disorders, topiramate may be viewed by providers as a way to address multiple disorders with one medication.

### Limitations

This study has several limitations, including a cross-sectional observational study design that was limited to patients treated in VHA facilities, which limits causal interpretation of findings and may not generalize to utilization patterns in other health-care systems. Another limitation is that persistence of treatment with topiramate was not examined, which may differ from our findings solely based on filling at least one prescription for topiramate; we cannot determine, for instance, whether a patient actually took the medication or whether there was treatment response or ill effect. In addition, there were several unmeasured variables, such as treatment motivation or the presence of other indications for topiramate, which may better explain the reported associations. Finally, the data used in this study did not permit us to determine if topiramate was specifically prescribed for AUD. In some cases, it may have been prescribed for some other common indications (e.g., migraine or seizure disorder), although 78% of the prescriptions for topiramate cannot be explained by these common uses. Further, we conducted several sensitivity analyses that accounted for several of these common indications and the results remained virtually unchanged.

## Conclusions

Veterans Health Administration facilities are monitored regarding the extent to which patients with AUD are receiving FDA-approved pharmacotherapy. Approximately 2% of the nearly 400,000 VHA patients with AUD were prescribed topiramate, and there was substantial facility-level variation in use. Several patient characteristics were associated with greater likelihood of topiramate prescription, including being female, younger age, diagnosis of alcohol dependence (versus alcohol abuse), treatment in both mental health and addiction specialty care, and having a psychiatric comorbidity. Not including topiramate in the quality improvement metrics appears to underestimate the extent to which VHA patients, at specific facilities and overall, are being prescribed pharmacotherapy for AUDs. We believe that the evidence is sufficient to consider topiramate an evidenced-based treatment for alcohol dependence. Therefore topiramate should be included in related quality measures and system monitoring metrics in VHA and elsewhere.

## Competing interests

The authors declare that they have no competing interests.

## Authors' contributions

AD participated in the study design, performed the statistical analysis, and helped to draft the manuscript. AG participated in its coordination and helped to draft the manuscript. AL participated in its coordination and helped to draft the manuscript. AH conceived of the study, participated in its design and coordination, and helped to draft the manuscript. All authors read and approved the final manuscript.
